# Smart HVAC Control in IoT: Energy Consumption Minimization with User Comfort Constraints

**DOI:** 10.1155/2014/161874

**Published:** 2014-06-18

**Authors:** Jordi Serra, David Pubill, Angelos Antonopoulos, Christos Verikoukis

**Affiliations:** Centre Tecnològic de Telecomunicacions de Catalunya (CTTC), 08860 Castelldefels, Spain

## Abstract

Smart grid is one of the main applications of the Internet of Things (IoT) paradigm. Within this context, this paper addresses the efficient energy consumption management of heating, ventilation, and air conditioning (HVAC) systems in smart grids with variable energy price. To that end, first, we propose an energy scheduling method that minimizes the energy consumption cost for a particular time interval, taking into account the energy price and a set of comfort constraints, that is, a range of temperatures according to user's preferences for a given room. Then, we propose an energy scheduler where the user may select to relax the temperature constraints to save more energy. Moreover, thanks to the IoT paradigm, the user may interact remotely with the HVAC control system. In particular, the user may decide remotely the temperature of comfort, while the temperature and energy consumption information is sent through Internet and displayed at the end user's device. The proposed algorithms have been implemented in a real testbed, highlighting the potential gains that can be achieved in terms of both energy and cost.

## 1. Introduction

The Internet of Things (IoT) paves the way for the connection of sensors, actuators, and other objects to the Internet, permitting the perception of the world, as well as the interaction with it, in an unprecedented manner. In addition, IoT will foster a huge number of new applications, for example, environmental monitoring, healthcare, and efficient management of energy in smart homes [1], potentially generating important economic benefits [2]. Actually, the US National Intelligence Council considers IoT as one of the six disruptive civil technologies with potential impact on US national power [3]. As a result, the concept of IoT, in terms of architectural aspects, protocol stacks, applications, and conceptual visions, has recently started to be studied [4–7].

Smart grid is considered as one of the main IoT applications and it has attracted a great interest during the last few years [1, 8, 9]. The smart grid is envisioned as the evolution of the current energy grid, which faces important challenges, such as blackouts caused by peaks of energy demand that exceed the energy grid capacity [10]. A proposed approach to alleviate this problem is to incentivize the consumers to defer or reschedule their energy consumption to different time intervals with lower expected power demand. These incentives are based on smart (or dynamic) pricing tariffs that consider a variable energy price [11]. For instance, in real-time pricing (RTP) tariffs, the price of the energy will be higher at certain time periods, where the energy consumption is expected to be higher, for example, during the afternoon or in cold days. Other types of smart pricing tariffs are critical-peak pricing (CPP) or time-of-use pricing (ToUP) [11–13]. Energy scheduling algorithms are the state-of-the-art methods to manage the energy consumption of loads within a smart pricing framework [11, 12, 14–16]. These techniques assume a specific smart pricing tariff and various time periods. For each of these time intervals, the scheduler determines the operational power of each appliance to minimize the energy consumption cost. It is worth mentioning that the appliances that can be controlled by the energy scheduler can be categorized into three classes: (i) nonshiftable, which do not admit any change on their consumption profile, (ii) time-shiftable, which tolerate postponing their operation, but not their consumption profile, and (iii) power-shiftable, whose operational power can be changed.

Regarding the power-shiftable loads, heating, ventilation, and air conditioning (HVAC) modules are considered as the most energy demanding appliances in home buildings [17, 18]. According to studies, they represent the 43% of residential energy consumption in the USA and the 61% in UK and Canada [18]. Apparently, the significant energy consumption of the HVAC systems, along with their direct influence on the user's well-being, highlights the necessity for effective HVAC management algorithms that reduce the power consumption in the home buildings, taking into account the end-user's comfort.

In this paper, we propose two HVAC energy scheduling methods in an IoT framework, where the users are able to interact remotely with the HVAC control system. In particular, the users may retrieve information about the temperature and the energy consumption at various spots of the building under control, while they are also able to remotely configure the temperature in given places. Our contribution can be summarized as follows.

- We propose a dynamic energy scheduler with comfort constraints (DES-CC), which considers both the smart pricing tariffs and the user's comfort, in order to select the most energy efficient configuration of HVACs that satisfies the user's needs. We formulate an optimization problem of HVAC control by predicting the temperature that a given set of HVAC modules would cause in different locations. We result in a boolean quadratic optimization problem which, although not convex, can be solved via an exhaustive search when the number of variables (i.e., HVAC modules in our case) is low. In case that a large number of HVAC modules are considered, semidefinite relaxation techniques can be applied [19].
- Taking into account the energy efficiency priority, we propose a dynamic energy scheduler with comfort constraints relaxation (DES-CCR), where the user relaxes their comfort constraints, allowing a higher degree of flexibility for the system to further reduce the energy consumption. In this case, the problem is reformulated and the user's comfort (i.e., temperature) is set as a penalty in the objective function instead of constraint.
- We have designed and developed a real testbed to evaluate the performance of the proposed algorithms, demonstrating the potential financial and energy gains that can be achieved.

The remainder of the paper is organized as follows.  Section 2 provides a brief review of the related work in this field.  Section 3 describes the general network architecture and the system model under study.  Section 4 introduces the two HVAC schedulers in a smart pricing and comfort constraint context.  Section 5 provides the description of the testbed and the experimental results. Finally, Section 6 concludes the paper.

## 2. Related Work

The energy cost management of HVAC systems has recently attracted the research attention. In [20], the energy cost is studied as a function of the parameters that control the air and water subsystems and an evolutionary programming method is proposed to save energy. Moreover, in [21], a dynamic threshold controls the energy consumption and it varies according to the user satisfaction, which also depends on a thermal model. However, neither [20] nor [21] explicitly consider a dynamic pricing cost. In [22], smart pricing is considered in the energy cost optimization, but the user comfort is not explicitly incorporated in the algorithm, as the authors consider that the HVAC is turned on/off when the indoor temperature is outside the margin of comfort. Recently, in [13, 18], both energy scheduling of HVAC under smart pricing and the user comfort are taken into account. In [13], Nguyen et al. propose the construction of a lookup table (LUT) of room temperatures that depends on (i) the past temperatures, (ii) the outdoor temperature, and (iii) the HVAC power. The authors claim that the LUT is built during a training period (that takes place only once) and permits to assess the temperature of comfort for a given operation of the HVAC energy scheduler. However, this heuristic approach seems hardly applicable in general scenarios. In [18], a linear energy cost function is considered, although quadratic or two-step piecewise linear functions are more common in practice [11], while user's comfort is measured only at a specific location. It is also worth noting that none of the aforementioned works considers an IoT framework.

Unlike [20–22], in our proposed energy scheduling methods, both the smart pricing tariffs and the user comfort are taken into account. Moreover, the temperature of comfort is measured at several building positions by different sensor nodes that form a wireless sensor network (WSN), thus providing a more accurate measure of comfort compared to [18]. Furthermore, compared to [13], we adopt a more analytical and less heuristic model to assess the user comfort in the HVAC energy cost optimization. In particular, our model considers the time varying nature of the real thermal conditions, without requiring a training period. Moreover, our model adaptively updates the past temperature measurements for each time period, whereas the model in [13] is only carried out once to construct the LUT for particular indoor and outdoor conditions. Finally, unlike most of the above references, our methods are validated in a real scenario.

## 3. Network Model

### 3.1. General Architecture

**Figure 1:**
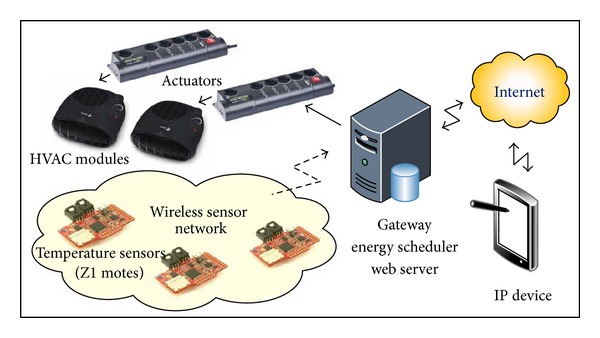
Overall architecture of the proposed HVAC energy scheduler in the IoT context.

Figure 1 presents the overall architecture which is used to evaluate the proposed energy schedulers in an IoT context. It consists of the following elements:

- a set of HVAC modules;
- a set of actuators that control the HVAC modules;
- a WSN, which sends measurements of temperature and energy consumption to a gateway;
- a gateway (GW) that incorporates the proposed energy scheduling methods and connects the local network to the Internet. That is, it contains a web server and a database to store data received at the GW from the WSN or the internet;
- an embedded IP device (e.g., tablet or smartphone) with an interface to interact with the HVAC energy scheduler. It also displays both the temperature and the energy consumption in the building measured by the WSN.

The functionality and flow of information of the proposed architecture is explained as follows. The temperature is measured at several locations by means of the WSN. Then, the measurements are periodically sent to the gateway, where the energy scheduling algorithm is implemented. This algorithm selects the combination of the active HVAC modules that minimizes the energy cost for given comfort constraints and energy price during a particular time period. These decisions are sent, through shell commands, to programmable surge protectors (actuators), which actuate the HVAC modules. The HVAC modules modify the room temperature according to the decisions taken by the energy scheduler.

**Figure 2:**
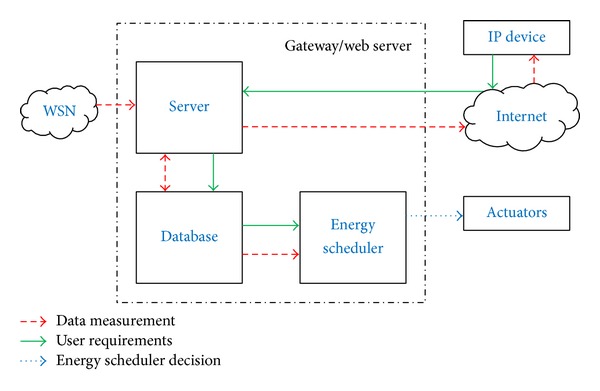
System model of the gateway.

Moreover, the gateway hosts a database to store the measurements of temperature and energy consumption. These measurements can be accessed by a remote Internet user. More specifically, they are displayed at the user's IP device, as the gateway implements a web server which manages the communication between the remote user and the local database. This is illustrated in more detail in Figure 2, where the connections between the most relevant blocks are shown. Furthermore, users are allowed to interact with the energy scheduler through their IP devices, by setting the upper and lower bounds of the temperature of comfort.

### 3.2. System Model

**Figure 3:**
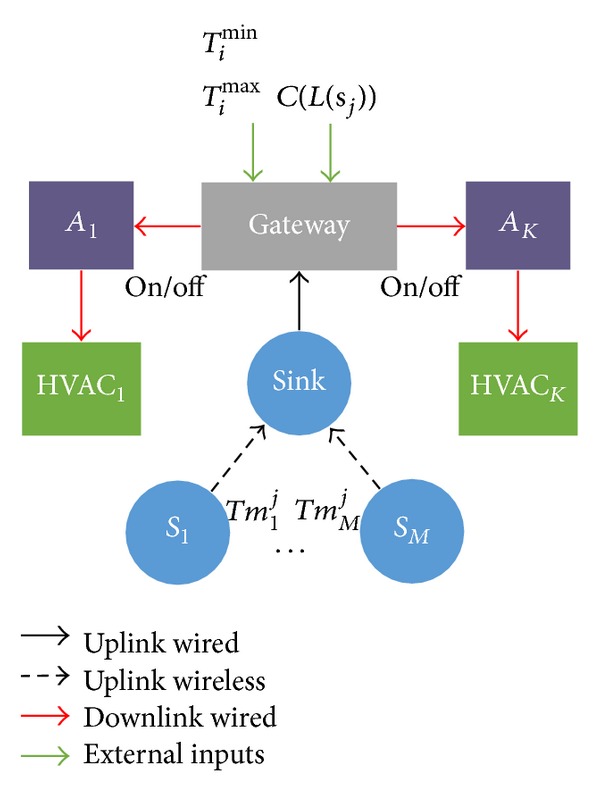
Detailed system model of the HVAC energy scheduler.

The system model for the proposed HVAC energy scheduler is depicted in more detail in Figure 3. In particular, the energy scheduler is implemented within the gateway and it interacts with the following modules. First, a WSN composed of *M* sensor nodes *S*_*i*_, 1 ⩽ *i* ⩽ *M*, which sense the temperature and transmit the measurements to the energy scheduler, through a WSN sink node. Second, a set of *K* HVAC modules that are controlled by the energy scheduler through a set of actuators *A*_*k*_, 1 ⩽ *k* ⩽ *K*. Moreover, the inputs that the energy scheduler requires are described as follows.

**Figure 4:**
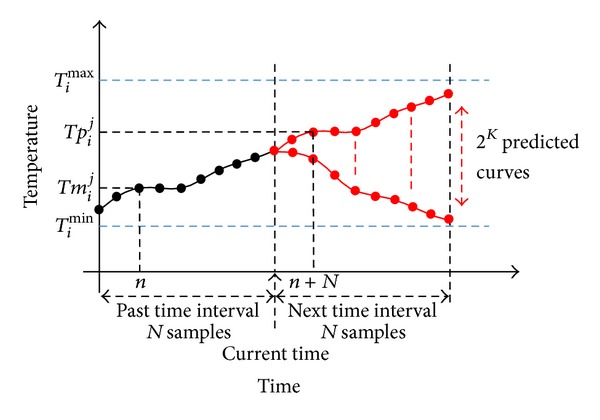
Prediction of temperature, a fundamental step of the energy scheduler to assess comfort in the future time interval.

(i) The measurements taken by the WSN nodes. For each time interval, *N* measurements are taken by each node when a given configuration of HVAC modules is turned on. These measurements are denoted by *Tm*_*i*_^*j*^(*n*) (as illustrated by the black curve in Figure 4), where 1 ⩽ *i* ⩽ *M* denotes the *i*th node and 1 ⩽ *j* ⩽ 2^*K*^ is the *j*th combination of HVACs turned on or off.
 (ii) The energy cost function *C*(*L*(**s**_*j*_)), which is specified by the energy provider and depends on the smart pricing tariff. According to [11], *C*(*L*(**s**_*j*_)) can be modeled as a quadratic function, that is,
  (1) $$\begin{array}{c} C\left(L\left(s_{j}\right)\right)=p_{1}{\left(L\left(s_{j}\right)\right)}^{2}+p_{2}L\left(s_{j}\right)+p_{3}, \end{array}$$
 where *p*_1_, *p*_2_, and *p*_3_ are parameters that the provider can dynamically vary in time. Moreover, *L*(**s**_*j*_) denotes the user's energy consumption for the **s**_*j*_ combination of HVAC modules turned on. In order to describe the expression for *L*(**s**_*j*_), let us define **P** ∈ *R* ^*K*×2^*K*^^ a matrix that contains, in its *j*th column, the energy consumption of each HVAC module for the *j*th combination of modules switched on/off. For instance, for *K* = 3, the matrix **P** is
  (2) $$\begin{array}{c} P=\left(\begin{array}{cccccccc} 0 & x_{1} & 0 & 0 & x_{1} & x_{1} & 0 & x_{1}\\ 0 & 0 & x_{2} & 0 & x_{2} & 0 & x_{2} & x_{2}\\ 0 & 0 & 0 & x_{3} & 0 & x_{3} & x_{3} & x_{3} \end{array}\right). \end{array}$$
 Moreover, **s**_*j*_ is a vector of all zeros except in the *j*th position that has value 1. Therefore, **P** **s**_*j*_ selects the energy consumption related to the *j*th combination of HVACs switched on and 1^*T*^(**P** **s**_*j*_) is the energy consumption related to that combination where 1 is a vector of ones of length 2^*K*^. Therefore, the total energy consumption for a given time period, denoted by *L*(**s**_*j*_), can be expressed as
  (3) $$\begin{array}{c} L\left(s_{j}\right)=L_{0}+1^{T}\left(Ps_{j}\right), \end{array}$$
 where *L*_0_ is the accumulated consumption and 1^*T*^(**P** **s**_*j*_) is the consumption in the current time interval decision.
 (iii) The constraints of temperature of comfort provided by the user. For the energy scheduler proposed in Section 4.1, this corresponds to the minimum and maximum allowed temperatures at the *i*th location of the room, which are denoted by *T*_*i*_^min⁡^ and *T*_*i*_^max⁡^ with 1 ⩽ *i* ⩽ *M*, respectively, since we assume that the user may specify the desired comfort at *M* different locations. For the energy scheduler proposed in Section 4.2, the comfort is specified by the objective temperatures *T*_*u*,*i*_, 1 ⩽ *i* ⩽ *M*, which the user would like to attain at the different *M* locations of the room, though they may allow some relaxation in order to further reduce the energy consumption.

To further clarify the operation of the proposed scheme, let us shed light on the temporal behavior of the energy schedulers and the role of the temperature constraints on it. Recall that the energy scheduler works in a time interval basis. At the end of each time interval (“current time” in Figure 4), the energy scheduler must make a new decision. That is, it must decide which HVAC modules, denoted by HVAC_*k*_ in Figure 3, will be active during the next time interval. In order to make this decision, the energy scheduler should predict which would be the temperature provoked by each configuration of HVACs. As there are *K* HVAC modules and we assume that they are either turned on or off, this corresponds to predict 2^*K*^ curves of temperature, as it is illustrated in Figure 4. These predicted temperatures are denoted by *Tp*_*i*_^*j*^(*n*), where *i* and *j* have the same meaning as for the case of *Tm*_*i*_^*j*^(*n*), explained above. Finally, on one hand, the DES-CC selects the configuration of HVACs that minimizes the energy consumption cost *C*(*L*(**s**_*j*_)) within the bounds of comfort, that is, *T*_*i*_^min⁡^ ⩽ *Tp*_*i*_^*j*^(*n*) ⩽ *T*_*i*_^max⁡^, while the DES-CCR selects the HVAC configuration that optimizes the tradeoff between being closer to the comfort temperatures *T*_*u*,*i*_ and saving energy. This selection is executed by the actuators that control the HVAC modules, which are denoted by *A*_*k*_ in Figure 3.

## 4. HVAC Energy Scheduling

### 4.1. Dynamic Energy Scheduler with Comfort Constraints (DES-CC)

Next, we present the first of the two proposed HVAC energy schedulers. To that end, this section is divided into four parts. First, we formulate the energy scheduler as a constrained optimization problem. Second, recall that, for each time interval, the energy scheduler must decide the combination of active HVACs to minimize the energy consumption cost and fulfill the constraints of temperature of comfort. To assess these constraints, the temperature provoked in the next time interval by each configuration of HVACs turned on or off should be predicted. Thereby, the second part deals with a thermal model that paves the way to predict the future temperatures. The third part specifies how to estimate the parameters of the prediction model thanks to the measurements of temperature of the past time interval. Finally, in the fourth part, we summarize the proposed DES-CC algorithm.

#### 4.1.1. Formulation of DES-CC as a Constrained Optimization Problem

The energy scheduler works in a time interval basis. When *N* samples of temperature have been collected from the WSN at the *M* controlled locations, the energy scheduler makes a new decision with respect to the state of the HVACs. Namely, for the next time interval, the energy scheduler selects the optimal configuration of active HVACs. This configuration, on the one hand, must minimize the energy consumption cost, while, on the other hand, it must respect the comfort constraints; that is, it should lead to predicted temperatures within the bounds of comfort. According to the definitions of the system model, this optimization problem may be formulated mathematically as

(4) minimizesj∈{0,1}2K×1 C(L(sj))subject  to Timin⁡⩽Tpimin⁡(sj), i∈[1,M]Timax⁡⩾Tpimax⁡(sj), i∈[1,M]1Tsj=1,

where *Tp*_*i*_^min⁡^(**s**_*j*_) and *Tp*_*i*_^max⁡^(**s**_*j*_) are the minimum and maximum predicted temperatures, respectively, at the *i*th location for the *j*th combination of HVAC modules turned on. Given the definition of *C*(*L*(**s**_*j*_)) in (1) as a quadratic function, the optimization problem (4) has the form of a quadratic programming, but that the optimization variable is boolean. Hence, it is a boolean quadratic programming problem. The problems of this class are nonconvex and, in general, they can be solved either by a fast method that finds a local solution or by a slower method that finds the global solution. In our framework, the number of HVAC modules *K* is low or moderate and the latter approach is preferred; for example, the branch and bound method [23] can be used. In order to proceed, we need to model the predicted temperatures *Tp*_*i*_^min⁡^(**s**_*j*_) and *Tp*_*i*_^max⁡^(**s**_*j*_).

#### 4.1.2. Model to Predict the Temperatures of the Future Time Interval

Regarding the predicted temperatures *Tp*_*i*_^min⁡^(**s**_*j*_) and *Tp*_*i*_^max⁡^(**s**_*j*_), they can be expressed as

(5) $$\begin{array}{c} Tp_{i}^{\min}\left(s_{j}\right)={\overset{{}^{\circ}}{q}}_{i}^{T}s_{j},\\ Tp_{i}^{\max}\left(s_{j}\right)={\dot{q}}_{i}^{T}s_{j}, \end{array}$$

where ${\overset{{}^{\circ}}{q}}_{i}$ and ${\dot{q}}_{i}$ are vectors that contain the minimum and maximum predicted temperatures, respectively, for each of the possible combinations of operating HVAC modules. To further clarify, let us define *Tp*_*i*_^*j*^(*n*); 2 ≤ *n* ≤ *N* the predicted temperature at the *n* time instant at the *i*th sensor for the *j*th combination of HVAC modules turned on, where 1 ⩽ *i* ⩽ *M* and 1 ⩽ *j* ⩽ 2^*K*^. Moreover, let *Tp*_*i*_^*j*^(*n*_min⁡_*j*__) and *Tp*_*i*_^*j*^(*n*_max⁡_*j*__) be the minimum and maximum temperatures among *Tp*_*i*_^*j*^(*n*); 2 ≤ *n* ≤ *N*. Then, ${\overset{{}^{\circ}}{q}}_{i}$ and ${\dot{q}}_{i}$ can be expressed as

(6) $$\begin{array}{c} {\overset{{}^{\circ}}{q}}_{i}^{T}=\left[Tp_{i}^{1}\left(n_{\min_{1}}\right),\ldots ,Tp_{i}^{2^{K}}\left(n_{\min_{2^{K}}}\right)\right],\\ {\dot{q}}_{i}^{T}=\left[Tp_{i}^{1}\left(n_{\max_{1}}\right),\ldots ,Tp_{i}^{2^{K}}\left(n_{\max_{2^{K}}}\right)\right]. \end{array}$$

In order to proceed, a model for the predicted temperatures is necessary. Intuitively, the current temperature is correlated with the past temperature and a given combination of HVACs turned on causes a change in temperature. Moreover, the temperature dynamics are rather linear (at least locally), as it will be shown below. Therefore, the following model is proposed for the temperature prediction:

(7) $$\begin{array}{c} Tp_{i}^{j}\left(n\right)=a_{i}^{j}Tp_{i}^{j}\left(n-1\right)+\gamma_{i}^{j},\;2\leq n\leq N, \end{array}$$

where *a*_*i*_^*j*^ and *γ*_*i*_^*j*^ model the relation with the past temperature and the change of temperature provoked by the *j*th combination of HVACs turned on, respectively. Observe that, in this expression, *a*_*i*_^*j*^ and *γ*_*i*_^*j*^ are unknown and must be estimated from the past measurements.

#### 4.1.3. Estimation of the Prediction Model Parameters

In order to estimate *a*_*i*_^*j*^ and *γ*_*i*_^*j*^ in (7), we assume that the past measurements follow a model like (7), corrupted by noise:

(8) $$\begin{array}{c} Tm_{i}^{j}\left(n\right)=a_{i}^{j}Tm_{i}^{j}\left(n-1\right)+\gamma_{i}^{j}+w_{i}^{j}\left(n\right),\;2\leq n\leq N. \end{array}$$

Note that the evolution of the temperature is considered to be linear in (7) and (8). This is a valid assumption at least for short periods, as the real experiments that we will present in Section 5 will highlight.

For the estimation of *a*_*i*_^*j*^ and *γ*_*i*_^*j*^, two situations are considered. In the first one, all the HVAC modules are switched off and, as a consequence, only *a*_*i*_^*j*^ must be estimated. To that end, least squares (LS) estimator is considered as no probabilistic assumptions regarding the data are needed. This estimator minimizes the LS error criterion, though it is not optimal in general [24]. Given (8), the LS estimation of *a*_*i*_^*j*^, denoted by ${\hat{a}}_{i}^{j}$ is given by

(9) $$\begin{array}{c} {\hat{a}}_{i}^{j}=x^{\#}y, \end{array}$$

where the symbol # denotes the pseudoinverse operator, which is defined as **x** ^#^ = (**x** ^*T*^ **x**)^−1^ **x** ^*T*^, and we define **x** = [*Tm*_*i*_^*j*^(1),…,*Tm*_*i*_^*j*^(*N*−1)]^*T*^ and **y** = [*Tm*_*i*_^*j*^(2),…,*Tm*_*i*_^*j*^(*N*)]^*T*^. The second situation is that some of the HVAC modules were switched on. In this case, an LS estimation is considered as well. Namely, let us denote by ${\hat{\gamma }}_{i}^{j}|{\overset{ˇ}{a}}_{i}^{j}$ the estimation of *γ*_*i*_^*j*^ conditioned to the knowledge of a past estimation of *a*_*i*_^*j*^, denoted by ${\overset{ˇ}{a}}_{i}^{j}$. Then, the LS estimation for ${\hat{\gamma }}_{i}^{j}|{\overset{ˇ}{a}}_{i}^{j}$ yields

(10) $$\begin{array}{c} {\hat{\gamma }}_{i}^{j}\;|\;{\overset{ˇ}{a}}_{i}^{j}=1^{\#}z, \end{array}$$

where 1 is a vector of ones of length *N* − 1 and **z** is given by

(11) z=[Tmij(2),…,Tmij(N)]T−aˇij[Tmij(1),…,Tmij(N−1)]T.

Finally, given the estimation of *γ*_*i*_^*j*^ in (10), we can update the estimation of *a*_*i*_^*j*^ as

(12) $$\begin{array}{c} {\hat{a}}_{i}^{j}\;|\;{\hat{\gamma }}_{i}^{j}=x^{\#}\tilde{y}, \end{array}$$

where $\tilde{y}={\left[Tm_{i}^{j}\left(2\right)-{\hat{\gamma }}_{i}^{j},\ldots ,Tm_{i}^{j}\left(N\right)-{\hat{\gamma }}_{i}^{j}\right]}^{T}$.

#### 4.1.4. Summary of the DES-CC Energy Scheduler

At this point, all the terms in the optimization problem under study, that is, (4), are specified. The procedure to implement the proposed energy scheduler for each time interval is summarized in Algorithm 1,

### 4.2. Dynamic Energy Scheduler with Comfort Constraints Relaxation (DES-CCR)

Despite its effectiveness and its obvious advantages, the proposed energy scheduling algorithm is completely focused on the temperature constraints, neglecting the price aspects of the problem. More specifically, although there could be time periods where the energy price increases, the energy scheduler switches on the same combination of HVAC modules, in order to respect the temperature constraints. However, in such cases, users might compromise their comfort preferences to decrease the energy consumption. In order to allow the user to have more flexibility in the energy consumption, a new energy scheduler will be presented in this section. This flexibility is implemented in terms of relaxing the temperature constraints to further reduce the energy consumption.

This new energy scheduler is formulated so that the user temperature constraints in (4) are skipped and they are incorporated as a penalty term in the objective function. Consequently, the new optimization problem can be written as

(13) minimizesj∈{0,1}2K×1 θC(L(sj))α+(1−θ)       ×∑i=1M∑n=2N||qiT(n)sj−Tu,i||2β,

where *C*(*L*(**s**_*j*_)) is the energy cost function, defined in (1). The vector **q**_*i*_^*T*^(*n*) is defined as

(14) $$\begin{array}{c} q_{i}^{T}\left(n\right)=\left[Tp_{i}^{1}\left(n\right),\ldots ,Tp_{i}^{2^{K}}\left(n\right)\right], \end{array}$$

and recall that *Tp*_*i*_^*j*^(*n*) is the predicted temperature at the *i*th location for the *j*th combination of HVACs modules turned on or off, see (7). Moreover, *α* and *β* are normalizing constants to adjust the values of the two terms in (13). Indeed, we set their value as

(15) $$\begin{array}{c} \alpha =C\left(L\left(s_{2^{K}}\right)\right)\\ \beta =\underset{s_{j}\in {\left\{0,1\right\}}^{K\times 1}}{\max}\overset{M}{\underset{i=1}{\sum }}\overset{N}{\underset{n=2}{\sum }}{||q_{i}^{T}(n)s_{j}-T_{u,i}||}^{2}, \end{array}$$

where *C*(*L*(**s**_2^*K*^_)) is the cost for all the HVAC modules turned on. The term *T*_*u*,*i*_ is the desired temperature that the user would like to maintain at the *i*th location of the room. Clearly, our reformulation balances the two optimization problems, that is, the energy cost minimization and the user comfort maximization. Note that the user comfort is defined as an Euclidean norm, but it can eventually be redefined with another distance measurement.

Finally, *θ* ∈ (0,1) is defined by the user according to their preferences. For example, in the extreme case, where *θ* = 0, the demand response algorithm will not consider any price and it will directly control the HVAC modules so that the desired temperature is reached. On the contrary, when *θ* = 1, the HVAC modules will always remain off. In this context, the users should set the *θ* value according to their preferences and experience. The DES-CCR energy scheduler is summarized in Algorithm 2.

## 5. Experimental Results 

In order to emulate the complete communication in an IoT framework, we have designed and developed a custom testbed that integrates the described architecture. In our experiments, we focus on a heating system, although the proposed algorithms apply in general HVAC systems. In this section, we describe the testbed platform and the experimental scenario, we define a baseline thermostat model, and, finally, we present the experimental results of our proposed algorithms.

### 5.1. Testbed Description and Experimental Setup

**Figure 5:**
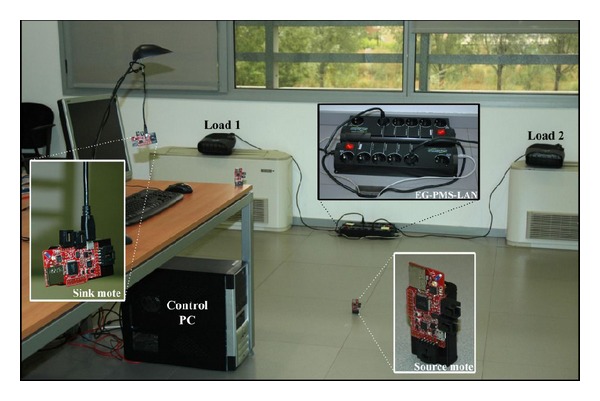
Z1 WSN mote.

The testbed has been deployed in a 50 m^2^ room within our research center facilities, as it is depicted in Figure 5. In our particular scenario, we consider three HVAC modules (i.e., *K* = 3) and two temperature sensor nodes (i.e., *M* = 2). The HVAC modules are distributed around the room, while the sensor nodes are placed in the middle of the room, monitoring the temperature and sending it to the sink mote every 30 second (i.e., *t*_*m*_ = 30 s). In addition, the samples received from each sensor are stored in a buffer at the control center and our algorithm applies every 10 samples (i.e., *N* = 10).

**Figure 6:**
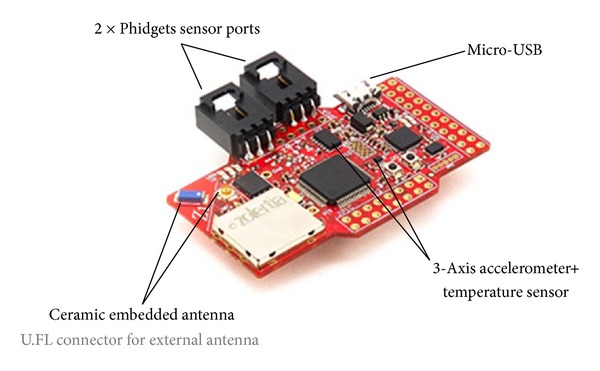
Overall platform detail.

Regarding the employed technology, the WSN nodes are Z1 motes by Zolertia (Figure 6). They are equipped with a second generation MSP430F2617 low power microcontroller, which features a 16-bit RISC CPU @16 MHz clock speed, a built-in clock factory calibration, an 8 KB RAM and a 92 KB flash memory. They also include the CC2420 transceiver, which is IEEE 802.15.4 compliant, operating at 2.4 GHz frequency band with a data rate of 250 kbps. The sensors support Contiki OS [25], an open-source operating system for the IoT, which connects tiny, low-cost, low-power microcontrollers to the Internet and supports IPv6 through 6LowPAN. It is worth noting that each mote can operate as either a source or a sink node. In particular, source nodes carry a TMP102 temperature sensor to monitor the target field, while the sink node receives and forwards the measured data to the gateway.

**Figure 7:**
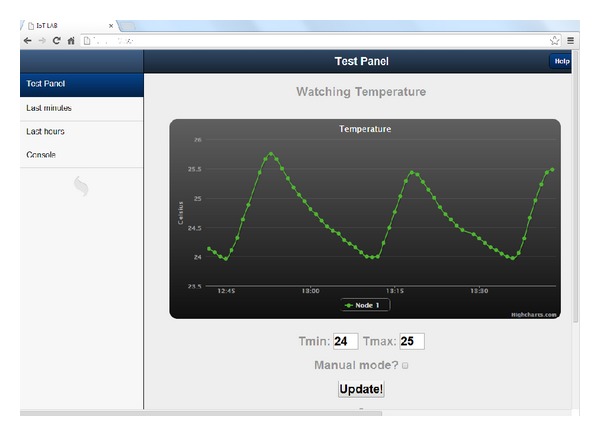
Google Chrome screenshot of the web application.

The gateway (an Ubuntu OS machine with MATLAB) implements the proposed algorithms and it is able to process the collected data. Furthermore, it connects the WSN to the Internet and acts as an application server, using Node.js and Sencha Touch. In particular, Node.js is a platform built on Chrome's JavaScript runtime for fast and scalable network applications.  Figure 7 shows a screenshot of the web application built on Node.js, which enables the user to interact with the energy scheduler through Internet. Regarding Sencha Touch, it is a high-performance HTML5 mobile application framework, which enables developers to build powerful applications for various operating systems, including iOS and Android. The actuators are programmable sockets which can be controlled remotely thanks to their IP addresses. These special sockets are a set of programmable local area network surge protectors (EG-PMS-LAN) by Energenie which are connected via Ethernet to the gateway. Finally, the HVAC modules are domestic heaters with a maximum power consumption of 2000 W.

### 5.2. Baseline Model

To evaluate the proposed algorithms and highlight the potential energy and cost gains that can be achieved, we adopt the traditional thermostat model as the baseline reference scenario. In this model, the aim is to maintain the average temperature of the room between a certain temperature range (i.e., [*T*_min⁡_, *T*_max⁡_]), predefined by the user. To that end, when the sensed temperature is above *T*_max⁡_ at the end of a time interval, all the heaters are switched off, while the heaters are switched on when the temperature falls below the *T*_min⁡_ threshold.

**Figure 8:**
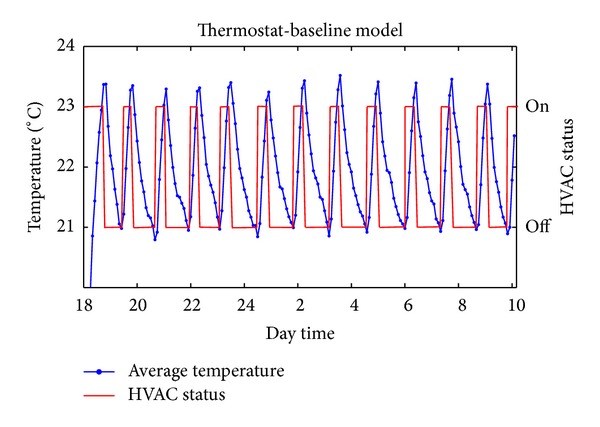
Experimental evaluation of the thermostat model.

Figure 8 illustrates the average measured temperature inside the room, where the heaters are controlled by the thermostat. In this particular case, we consider *T*_min⁡_ = 21°C and *T*_max⁡_ = 23°C. As it can be seen in the figure, the thermostat algorithm is able to maintain the average temperature of the room between the desired margins during 16 hours. However, it is worth noting that, despite its proper behavior, the particular model is not cost efficient, as all HVAC modules work simultaneously, consuming a total power consumption of 6000 W. In the following sections, we evaluate our proposed methods, demonstrating that they can reduce the electrical cost with respect to the baseline approach.

### 5.3. Experimental Evaluation of the DES-CC in (4)

Several real experiments have been carried out to assess the performance of the DES-CC algorithm, proposed in (4). In this case, the pricing parameters in (1) are *p*_1_ = 0.003 and *p*_2_ = *p*_3_ = 0 euros, which are possible values according to [11]. The temperature bounds in (4) have been set to *T*_1_^min⁡^ = *T*_2_^min⁡^ = 21°C and *T*_1_^max⁡^ = *T*_2_^max⁡^ = 23°C, respectively.

**Figure 9:**
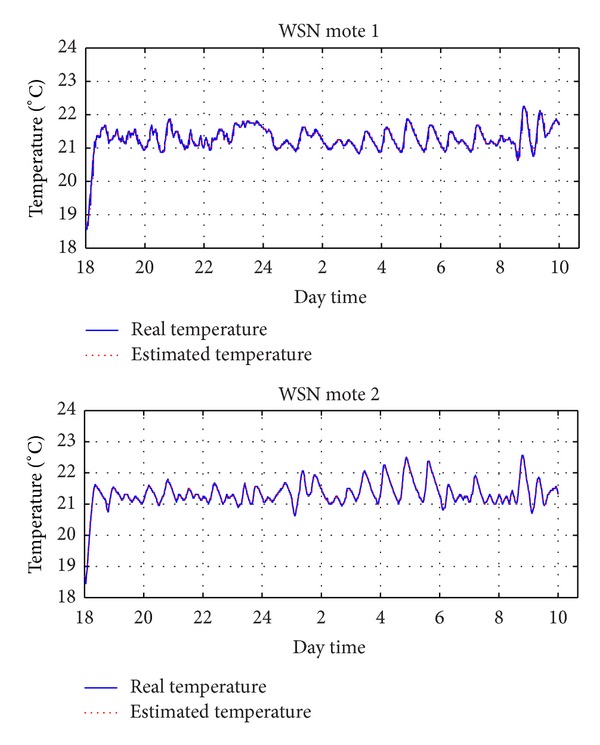
Real and estimated temperature using DES-CC.

Figure 9 plots the variation of both the measured and the estimated temperature (using (8)–(12)) in the room during our experiments. As it can be noticed, the error between the estimated and the real temperature is negligible, something that proves the accuracy of the proposed estimation model. In addition, the DES-CC guarantees the proper operation of the system, as the temperature varies between the desired range of 21°C and 23°C most of the time, with very few exceptions due to prediction errors. In the same figure, it can be also seen that the temperature remains closer to the lower part of the permitted range (i.e., 21°C), since the outcome of the proposed method provides a combination of switched on heaters that minimizes the energy consumption, satisfying a minimum acceptable temperature. Indeed, compared to the temperature variation in the baseline scenario (Figure 8), DES-CC maintains the temperature more stable and in the lower part of the allowable region, intuitively implying lower cost.

**Figure 10:**
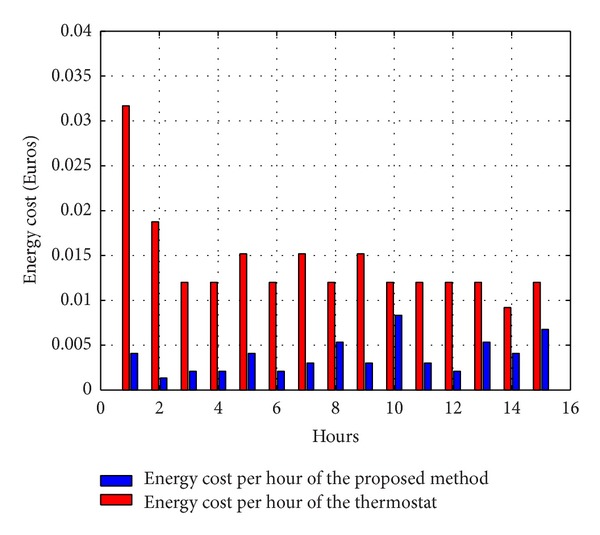
Energy consumption cost comparison between thermostat and DES-CC.

Figure 10 depicts the financial operation cost gains that can be achieved by DES-CC compared to the baseline thermostat approach. As it can be observed, the proposed energy scheduler significantly reduces the energy cost, leading to a total save of 7.19 euros/month.

### 5.4. Experimental Evaluation of the DES-CCR in (13)

A set of experiments have been carried out for the evaluation of the DES-CCR in (13). Let us recall that DES-CCR relaxes the temperature constraints by including the constraints as a penalized term in the objective function. As a result, compared to DES-CC, this method is more flexible with respect to real time pricing tariffs. More specifically, DES-CC seeks a combination that minimizes the energy cost with respect to a minimum allowable temperature. Consequently, although the energy cost may change during time, the heater combination selected by DES-CC is the same, due to the strict temperature constraint. On the other hand, DES-CCR allows the user to further reduce the energy consumption at the cost of being outside the range of temperature of comfort. In this case, to highlight the flexibility of DES-CCR, we have set a periodically variable value of *p*_1_, which alternates between *p*_1_ = 0.009 and *p*_1_ = 0.003 euros every thirty minutes. Moreover, the desired temperature has been set to *T*_*u*,*i*_ = 22°C.

**Figure 11:**
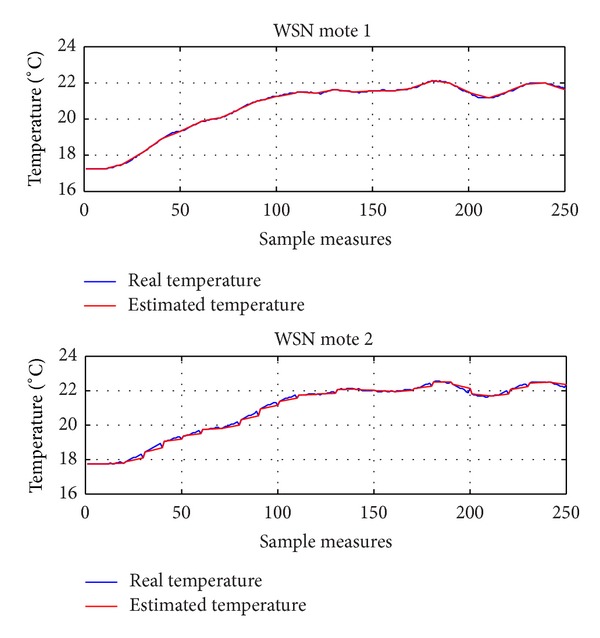
Real and estimated temperature using DES-CCR (*θ* = 0.2).

**Figure 12:**
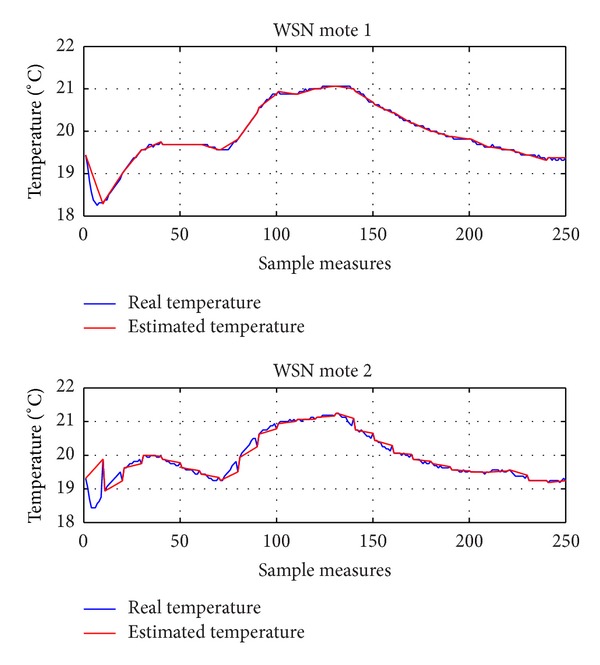
Real and estimated temperature using DES-CCR (*θ* = 0.5).

Figures 11 and 12 depict the temperature variation in two different cases, where the users give low (*θ* = 0.2) and high (*θ* = 0.5) priority, respectively, to reduce of the energy consumption. In particular, in Figure 11 (*θ* = 0.2), the achieved temperature is very close to the desired *T*_*u*,*i*_. On the other hand, in Figure 12, we assume *θ* = 0.5, which is a more adapted value to the pricing policy, as it corresponds to a user that permits a relaxation of the difference between the real and the desired temperature to reduce the energy cost. This fact implies higher energy consumption in low cost zones and lower energy consumption in high cost periods, sacrificing though the user's comfort. Therefore, the experiments confirm that the real temperature is close to *T*_*u*,*i*_ when the energy cost is lower (i.e., between samples 60 and 120), while there is a noticeable temperature drop, which corresponds to lower energy consumption.

## 6. Concluding Remarks

This paper has dealt with the energy consumption management of HVACs, for a given smart pricing tariff and users' comfort constraints. Moreover, the integration within the IoT framework has been studied. To that end, we developed a real testbed consisting of (i) heaters, (ii) sensor nodes that measure the temperature, and (iii) a gateway, which provides connection to the Internet and includes a web application that permits the interaction with the user through Internet. Moreover, the gateway implements the algorithms that control the energy consumption. Regarding the proposed methods, first, we devised an energy scheduler that optimizes the energy cost in a time interval basis, for a given energy price tariff and for a given set of temperature of comfort constraints that are associated with different locations inside a room. Then, we proposed a more flexible energy scheduler, which relaxes the temperature constraints to further reduce the energy consumption. Namely, a new objective function has been considered, which consists of a convex combination of the energy cost and a penalty term that reflects the comfort. This permits to consider both the case where the user is very concerned with the comfort and the case where he allows relaxing the comfort constraint to further reduce the energy consumption. Experimental evaluations have been carried out in an isolated room, validating our proposals and highlighting their potential benefits.

## Figures and Tables

**Algorithm 1:**
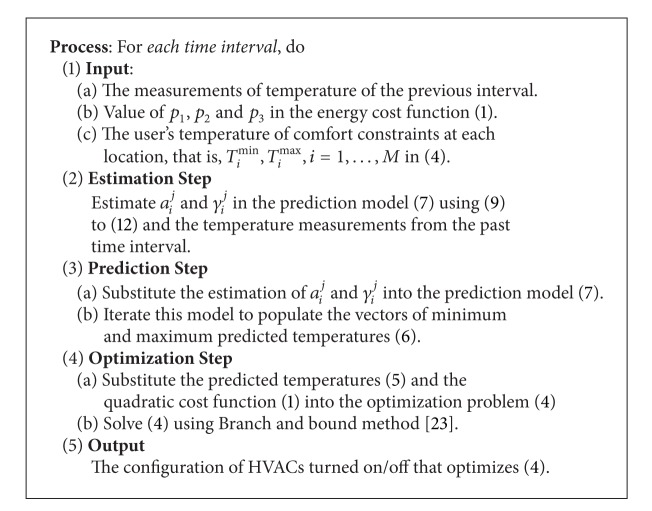
**Algorithm 1: **Dynamic energy scheduler with comfort constraints (DES-CC).

**Algorithm 2:**
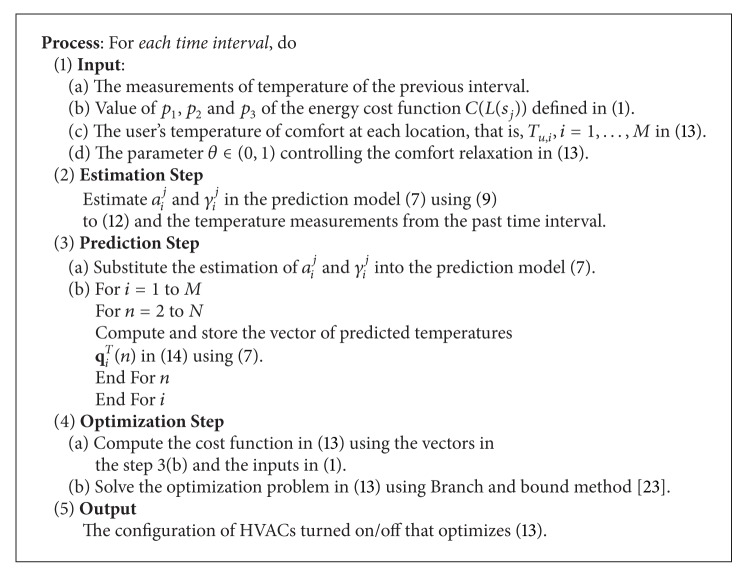
**Algorithm 2: **Dynamic energy scheduler with comfort constraints relaxation (DES-CCR).
